# Learning from the kidney: improving heart recovery from hypothermia

**DOI:** 10.3389/fcvm.2026.1930065

**Published:** 2026-09-21

**Authors:** Casey Kraft, John C. Bischof, Erik B. Finger

**Affiliations:** 1 Department of Biomedical Engineering, University of Minnesota, Minneapolis, MN, United States; 2 Department of Mechanical Engineering, University of Minnesota, Minneapolis, MN, United States; 3 Institute for Engineering in Medicine, University of Minnesota, Minneapolis, MN, United States; 4 Department of Surgery, University of Minnesota, Minneapolis, MN, United States

**Keywords:** acidosis, CPA toxicity, cryopreservation, hypothermia, ischemia reperfusion injury (IRI), metabolism, organ preservation, sodium overload

## Abstract

Extending donor organ preservation times would substantially improve patient outcomes by widening safety margins and enabling better donor-to-patient matching. While clinical 4°C static storage typically preserves donor kidneys for 18–24 h, donor hearts are limited to ∼4 h. This clinical disparity is traditionally attributed to the higher baseline metabolic demand of cardiac tissue. This narrative review challenges this conventional assumption by comparing the distinct metabolic profiles of the heart and kidney at both 37°C and 4°C. We propose two mechanisms to explain how kidneys maintain robust functional recovery despite a much steeper decline in ATP concentration during hypothermic storage: (1) a significantly higher ATP consumption rate in hypothermic kidneys compared to hearts, and (2) a lower susceptibility to and degree of cold-induced tissue acidosis in the kidney. Building on these pathways, we outline targeted therapeutic interventions, such as inhibition of the Na^+^/H^+^-exchanger, to mitigate downstream sodium and calcium overload in cardiac tissue. Finally, we advance acidosis as a salient concern in organ cryopreservation, offering a fresh mechanistic framework (the acidosis hypothesis) to alleviate non-specific cryoprotective agent (CPA) toxicity and enhance heart tolerance during hypothermic CPA exposure.

## Introduction

In organ transplantation, the risk of irrevocable damage at extended storage time motivates clinicians to transplant the donor organ as soon as possible. From 2010 to 2018, the average storage time for donor hearts was 3 h (1). Yet, by safely extending this storage time, patient survival could be improved. For example, clinicians would have more time to conduct donor heart size matching, a process that is currently deprioritized despite the known detrimental effect of transplanting undersized or oversized hearts (2). Additionally, extending organ storage time would improve clinician quality of life given that ∼25% of all kidney transplants occur between 8 PM and 8 AM (3). One approach to extend organ preservation time is normothermic machine perfusion (4), though the higher upfront cost of normothermic perfusion systems [e.g., $65,000 (5)] combined with recent advancements on cold storage methods (6) motivates further work into understanding and improving organ preservation using cold static storage or cold perfusion storage. In this article, heart and kidney responses to hypothermia are compared to better understand the differences between these organs and to inform future cold storage strategies.

In clinical organ transplantation using 4°C static storage, the storage time guideline is ∼4 h for the heart (7) vs. 18–24 h for the kidney (8). One reason the storage time guideline is shorter for hearts than for kidneys is that transplanted hearts need to recover healthy function immediately, whereas transplanted kidneys can gradually recover function without compromising patient survival, given the option of dialysis (9). Another factor may be that the heart is more sensitive to cold ischemia than the kidney, though compelling evidence to support this idea is lacking. In our review of the literature, no published study compared functional recovery following cold static storage of hearts vs. kidneys under the same conditions, though existing data are suggestive that each organ is similarly sensitive to hypothermia. For example, rabbit hearts recovered ∼40% of function (left ventricular systolic pressure) following 24 h of cold storage (10), which is similar to the result of a different study in which rat kidneys recovered ∼40% of function (glomerular filtration rate) following 24 h of cold storage (11). Interestingly, despite this similar outcome, the metabolic profile during cold ischemia differs substantially between the heart and the kidney. In our review of the literature, the sole identified cold storage study that compared the metabolism of hearts vs. kidneys found that, under identical conditions of 4°C static storage in UW solution, rabbit kidney ATP concentration dropped to one-third of initial after 1 h at 4°C whereas rabbit heart ATP concentration was preserved after 6 h at 4°C and only dropped to one-third of initial after 24 h at 4°C (Figure 1A) (10).

**Figure 1:**
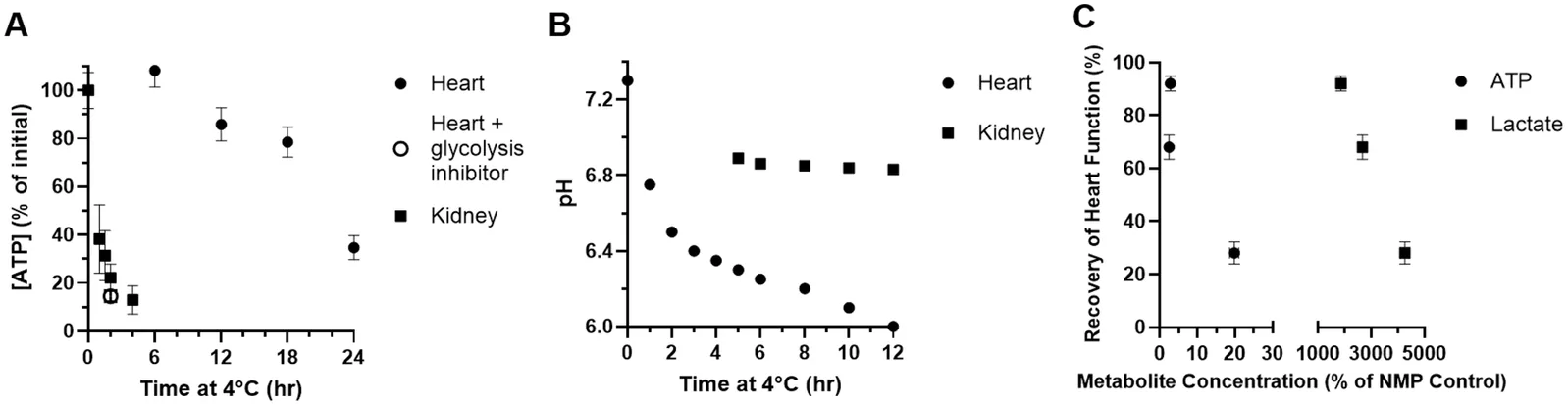
Heart vs. kidney metabolism during hypothermic storage **(A,B)** and the correlation of low [lactate] with heart recovery from ischemia **(C) (A)** ATP concentration during rabbit organ 4°C static storage in UW. The glycolysis inhibitor was 3 mM iodoacetate. These data (μmol ATP/g protein) were obtained from a study originally published elsewhere (10). **(B)** pH comparison during 4°C static storage using data from two studies with different methods. Heart myocardial pH data were collected using a micro-pH electrode implanted into porcine myocardium and originally published elsewhere (4). Kidney cytosolic pH measurements were collected using nuclear magnetic resonance on human kidneys and originally published elsewhere (40). **(C)** Rat heart recovery of function following 30 min of ischemia at 37°C. Function was calculated as the product of ventricular developed pressure and heart rate. Each value for functional recovery represents the average of one treatment group, which involved three different pre-ischemia perfusion conditions (10 min oxygenated, 10 min anoxic, and 15 min anoxic) and therefore different levels (μmol/g dry) of ATP and lactate in the tissue vs. the normothermic machine perfusion (NMP) control after ischemia. These data were replotted from tabulated values originally published elsewhere (43).

This hypothesis-generating article explores how kidneys can recover from cold ischemia at least as well as hearts despite their substantially lower ATP concentration during cold storage. In the aforementioned article on ATP concentration in cold-stored kidneys vs. cold-stored hearts, Stringham et al. advanced a twofold explanation. First, the kidney has a lower energy demand than the heart during 37°C reperfusion. Second, the ATP concentration limit for the kidney is much lower than that of the heart because only the heart can undergo irreversible contracture upon 37°C reperfusion (10). If true, this twofold explanation motivates an ATP-focused approach for extending organ preservation time.

In this hypothesis-generating review, evidence is presented that challenges and expands this understanding. First, we present our literature review comparing kidney and heart metabolism at both 37°C and 4°C. Second, we offer an explanation of acidosis to supplement the existing ATP-limit explanation for why kidneys can recover from cold ischemia at least as well as hearts. In light of this dual understanding of the importance of both ATP consumption and low acidosis, we then provide research strategies to improve heart recovery from hypothermia. As such, one application of this article is to provide potentially beneficial research directions for extended cold storage time.

A further application of this article is organ cryopreservation, an approach our group and others are pursuing to extend organ preservation time to multiple years (12). With this extra time, clinicians could perform extensive donor-to-patient matching and immune tolerance induction protocols—both of which can require weeks or more to complete but are expected to greatly improve outcomes for organ recipients—thereby better preparing patients for surgery (13). However, while our group has achieved repeatable cryopreservation success in rat kidneys (14), we have not been able to match these results in the heart (15). Based on our findings of low heart tolerance to the cryoprotective agent (CPA) exposure phase of our cryopreservation protocol (16), we have identified increasing heart tolerance to CPA as a critical step toward achieving cryopreservation success in the heart. Given that hypothermic temperature (e.g., 4°C) may help avoid CPA toxicity (17), our published heart CPA exposure protocols involved hypothermic temperature (15, 16) and therefore may benefit from a targeted strategy to address heart hypothermia injury. Additionally, cellular damage pathways involving oxidative stress and calcium overload are triggered by both CPA (18) and hypothermia (19), motivating investigation into the mechanisms of CPA toxicity and a therapeutic approach that targets any shared mechanistic causes of hypothermia injury and CPA injury. The leading mechanistic understanding of CPA toxicity is the qv* hypothesis (20), which is supported by data that some view as suggestive, albeit in need of more evidence to be convincing of the truth of the hypothesis (18). In this article, the acidosis hypothesis of CPA toxicity is advanced in an effort to improve our current understanding.

## Background

This section contains our literature review which was focused on organ-specific differences in metabolism to answer the research question how kidneys can recover from cold ischemia at least as well as hearts despite their substantially lower ATP concentration during cold storage.

### Metabolism at 37°C

Under normal physiological conditions, the kidney and the heart have a similarly high metabolic rate. According to data on humans at rest, the energy consumption rate is 440 kcal/kg organ/day for both the kidney and the heart, vs. a substantially lower rate for all other tested organs, such as 240 kcal/kg/day for the brain (21). This finding of the kidney's metabolic rate exceeding that of the brain may be particularly surprising, and therefore, further evidence is presented. In a study analyzing oxygen consumption rate across multiple tissues, though not cardiac tissue, kidney cortical slices were found to have a higher metabolic rate than brain cortical slices across 8 of 9 tested species (22). As evidenced by electron micrographs, these oxygen consumption data correspond with a higher mitochondrial density (% of cellular volume) in the kidney than in the brain (23).

While the total amount of energy consumed by the heart and kidney is similar, the utilization of this energy is different between the heart and the kidney. At 37°C, a greater percentage of heart metabolism involves mitochondrial ATP generation (via oxidative phosphorylation) than kidney metabolism. Key evidence for this includes higher mitochondrial density (% of cellular volume) and higher mitochondrial membrane surface area (per cm^3^ of tissue) in the heart than in the kidney (23). Further, the average heart mitochondrion is more metabolically active than the average kidney mitochondrion. In a study of mitochondria isolated from rat heart ventricles and rat kidney outer medulla, it was found that heart mitochondria exhibit a State 3 oxygen consumption rate >3 times that of kidney mitochondria (24). Note that the heart's higher steady-state ATP concentration than the kidney—5.5 (25, 26) vs. 1.4 μmol/g wet weight (27)—does not on its own entail that the heart has a higher ATP production rate than the kidney; a steady-state value of 5.5 μmol ATP/g wet weight could be achieved by either a low ATP production rate or by a high ATP production rate (given that ATP consumption rate equals ATP production rate at steady-state).

The heart's high rate of ATP generation via oxidative phosphorylation corresponds with its contractile function. This is because cellular ATP utilization is compartmentalized, with ATP generated from glycolysis tending to be used for ion balance (membrane pumps) and ATP generated from oxidative phosphorylation tending to be used for other cellular functions (e.g., actin-myosin contraction). Key evidence for this includes a study in which perfused rabbit septa exhibited worse contraction function (40% vs. 80% of initial) during inhibition of oxidative phosphorylation than during inhibition of glycolysis. This same study found that extracellular potassium concentration was better maintained (rising by 10% vs. 50%) during inhibition of oxidative phosphorylation than during inhibition of glycolysis (28). From this, it should be understood that glycolysis is not simply a backup process of ATP generation whose only use is during periods of hypoxia. Instead, a certain degree of glycolytic flux during 37°C normoxia is essential for healthy cellular function.

Accordingly, the kidney's function of ion balance corresponds with its relatively high percentage of ATP generation via glycolysis. Especially in regions of the kidney exposed to low oxygen, glycolysis provides an essential source of ATP to membrane pumps (especially the Na^+^/K^+^-ATPase) (29).

If not properly balanced, ATP consumption via glycolysis would result in local or systemic acidosis (lower pH). This is because the reaction ATP → ADP + P*_i_* is H^+^-generating (pH-lowering) and therefore needs to be balanced by a H^+^-consuming (pH-raising) process such as oxidative phosphorylation. ATP production via glycolysis, however, is pH-neutral, and therefore the entire process of glycolytic ATP production and consumption is acidifying (pH-lowering). This can be offset by the process of ATP production by oxidative phosphorylation and ATP consumption via gluconeogenesis, which is net alkalizing (pH-raising) (30).

In healthy systemic 37°C metabolism, certain organs can be classified as H^+^ sources, whereas others can be classified as H^+^ sinks. Whereas some organs, such as the heart, use lactate (the product of glycolysis) as an energy source, other organs, such as the kidney and liver, use lactate as a critical gluconeogenic precursor (31). Due to its negligible gluconeogenesis activity, the heart is therefore a H^+^ source, whereas the liver, due to its high gluconeogenesis activity, serves as the major systemic H^+^ sink. The kidney is of unclear classification due to its substantial glycolytic activity and, in some contexts, substantial gluconeogenic activity. During periods of acidosis, the kidneys can account for half of all systemic glucose production (via gluconeogenesis) with the liver producing the remainder (32–34).

### Metabolism at 4°C

During hypothermia, whether or not oxygen is present, cellular metabolism shifts away from oxidative phosphorylation and toward glycolysis. At <15°C, the decline in oxidative phosphorylation flux becomes particularly pronounced, presumably due to a mitochondrial membrane phase transition (35). In our review, one study was identified which compared metabolic parameters (ATP concentration) between the heart and the kidney during hypothermia using the same methods. Using 4°C static storage of rabbit organs, this study—which would benefit from replication—found that kidney ATP concentration was 30% of initial after 1 h at 4°C whereas heart ATP concentration was preserved at 100% of initial for the first 6 h at 4°C and only declined to 30% of initial after 24 h at 4°C (Figure 1A) (10).

There are two explanations for the sharper ATP concentration drop in the kidney compared with the heart at 4°C. The first is that the kidney consumes more ATP than the heart at 4°C. The second, which is not mutually exclusive with the first, is that the heart produces more ATP than the kidney at 4°C. To conclusively evaluate each explanation, data on ATP consumption and production rates for each organ are needed. However, while data on ATP production rate (μmol/s/g dry weight) have been collected for the heart at 37°C (36), to our knowledge no study has measured ATP consumption or production rates in the heart or the kidney during hypothermia. Therefore, the subsequent analysis (discussion section) presents suggestive evidence to interpret the difference between the heart and kidney at 4°C and to inform future work.

## Discussion

In this section we advance two hypotheses (Explanation 1 and Explanation 2) for why kidneys can recover from cold ischemia at least as well as hearts despite kidneys' substantially lower ATP concentration during cold storage (Figure 2).

**Figure 2:**
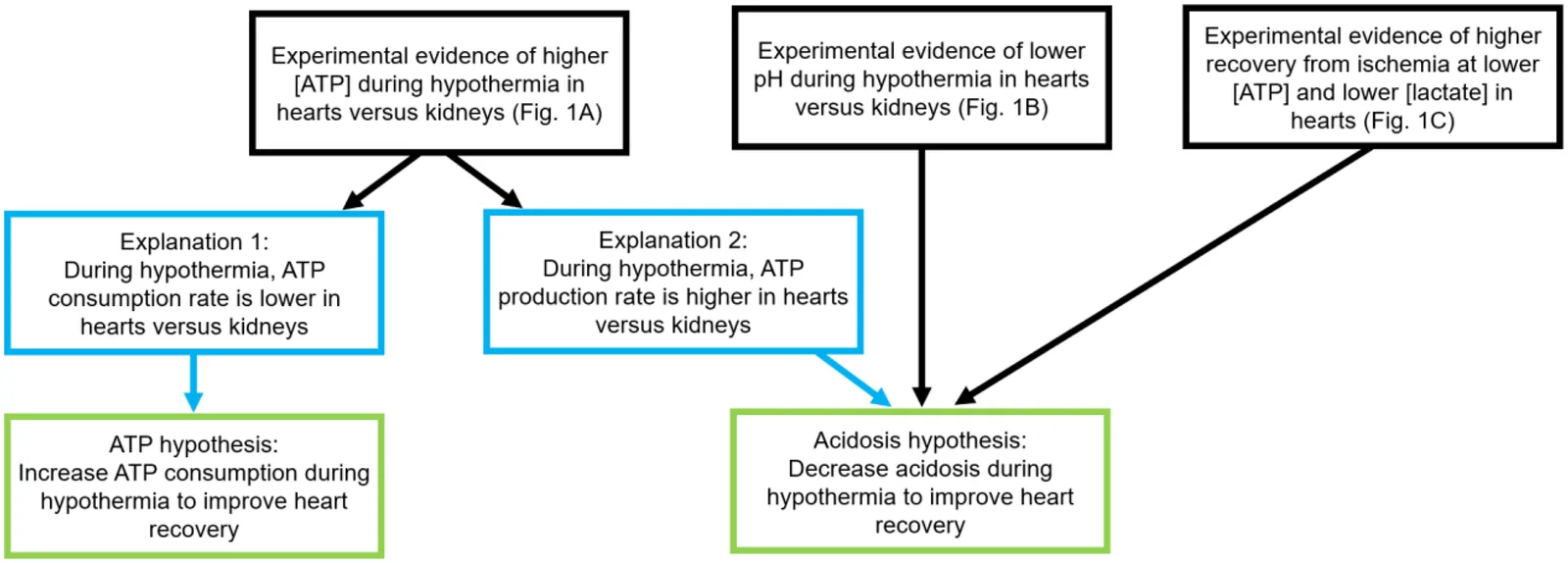
Learning from the kidney to generate hypotheses for improved heart preservation.

### Explanation 1: the heart has a substantially lower ATP consumption rate than the kidney at 4°C

This proposition (which is not established knowledge) would explain why ATP concentration declines more sharply for the kidney than the heart at 4°C (Figure 1A) (10) and is consistent with many efforts in the field (e.g., research on preserving ATP during storage). An argument for this proposition is as follows:

Premise 1: At 37°C, the ATP consumption rate due to actin-myosin contraction is higher in the heart (>>0 μmol ATP/s) than in the kidney (0 μmol ATP/s)
Premise 2: At 4°C, 0% of heart ATP consumption is due to actin-myosin contraction
Premise 3: At 37°C, the ATP consumption rate due to membrane pumps is higher in the kidney than in the heart
Premise 4: As temperature drops from 37°C to 4°C, membrane pump ATP consumption rates decline to a similarly low, nonzero percentage of their baseline activity in both organs
Conclusion: Consequently, at 4°C, the absolute ATP consumption rate remains higher in the kidney than in the heart

Premise 3 and Premise 4 are uncertain propositions that may require yet-to-be-collected data on the ATP consumption rate to be conclusively evaluated. Nonetheless, Premise 4 is supported by the approximately uniform effect of declining temperature on declining reaction rates (19), and Premise 3 is supported by measurements of higher Na^+^/K^+^ pump density in kidney cells than cardiomyocytes as calculated in studies using [^3^H]-ouabain to bind Na^+^/K^+^ pump molecules. In nephron segments dissected from collagenase-treated rabbit kidneys, there was a range of 3–50 million Na^+^/K^+^ pump molecules per cell across various segments of the nephron, with an average density of ∼20 million Na^+^/K^+^ pump molecules per kidney cell (37). In embryonic chick heart cells, the density was ∼10× lower at 2.2 million Na^+^/K^+^ pump molecules per cardiomyocyte (38), a difference that would cause significantly lower ATP consumption in the heart than the kidney if Na^+^/K^+^ pump turnover rate (molecules of ATP consumed per second) is not substantially different between the heart and the kidney. In a study using guinea pig ventricular myocytes, the maximum Na^+^/K^+^ pump turnover rate for the heart was calculated at 81 1/s (39). This is not substantially different than the kidney max Na^+^/K^+^ pump turnover rate, which was calculated at 33 1/s in a rabbit model (37). Therefore, Premise 3 is reasonable, though the limited evidence presented here would be more compelling if each heart vs. kidney parameter value was determined in a single study using the same methods.

An implication of this understanding is that to improve heart preservation time at 4°C, heart ATP production and consumption at 4°C should be increased (Figure 2). However, this approach conflicts with evidence that heart ATP production and consumption at 4°C are already substantial without any interventions. In the aforementioned study on 4°C static storage of rabbit organs, heart ATP concentration remained at 100% of initial after 6 h at 4°C unless a glycolysis inhibitor was used, in which case heart ATP concentration dropped to 15% of initial after 2 h at 4°C (Figure 1A) (10). This demonstrates that heart consumption of glycolytic ATP is already substantial at 4°C, raising skepticism about whether a lack of ATP consumption at 4°C is as much a concern for the heart as it is for the kidney.

### Explanation 2: the heart has a substantially higher ATP production rate than the kidney at 4°C

The second explanation (which is not established knowledge) for the sharper ATP concentration decline in kidneys than in hearts (Figure 1A) (10) is that the ATP production rate (via glycolysis) is lower in the kidney than in the heart at 4°C. An argument for this proposition is as follows:

Premise 1: During 4°C static storage, heart ATP concentration is lower in the presence of a glycolysis inhibitor (∼10% of initial ATP concentration after 2 h) than in the absence of a glycolysis inhibitor (∼100% of initial ATP concentration after 2 h) (Figure 1A)
Intermediate conclusion: During 4°C static storage, glycolysis is the major ATP production pathway in the heart
Premise 2: The process of ATP production via glycolysis (which is pH-neutral) and ATP consumption (which is acidifying) is net acidic (i.e., glycolysis enables a decrease in pH)
Premise 3: During 4°C static storage (in the absence of a glycolysis inhibitor), intracellular pH is lower in the heart than in the kidney
Conclusion: During 4°C storage, the absolute ATP production rate is higher in the heart than in the kidney

Of these premises, Premise 3 is not established knowledge and may most benefit from further study. Existing evidence in support of Premise 3 is that, during 4°C static storage of pig hearts, pH, as measured with an implanted electrode, fell to 6.8 during the first hour of 4°C storage and continued to decline to 6.0 after 12 h of 4°C storage (Figure 1B) (4). In contrast, during 4°C static storage of human kidneys, pH, as measured with nuclear magnetic resonance, was relatively stable from 5 to 12 h at 4°C, with a much higher value of 6.8 after 12 h of 4°C storage (Figure 1B) (40). A key limitation of this analysis is that pH was measured across disparate studies using fundamentally different methodologies—specifically, invasive microelectrodes in porcine models vs. noninvasive nuclear magnetic resonance (NMR) in human tissue. This variance highlights a critical gap in the preservation literature: a controlled, head-to-head study of cold ischemia-induced intracellular pH kinetics in both organs, using identical measurement modalities such as ^31^P-NMR spectroscopy, is urgently needed to validate these trends. For example, the study design could be *N* = 8 porcine hearts and *N* = 8 porcine kidneys stored ischemic at 4°C for 0–12 h in UW assessed continuously for intracellular pH using the non-invasive method of ^31^P-NMR spectroscopy with biopsies collected every 1–2 h for further data on ATP concentration and lactate concentration.

The validity of the reasoning step from Premise 3 (a difference in intracellular pH) to the advanced hypothesis (a difference in ATP production rate) depends on the direction and magnitude of factors other than glycolysis that influence intracellular pH. For example, the kidney can offset acidosis via gluconeogenesis whereas the heart cannot (32–34). Therefore, collecting ATP dynamics data (μmol ATP/s/g dry weight) would be clearest for evaluating whether the discrepancy in hearts vs. kidneys at 4°C (Figure 1A) (10) is due to Explanation 1 (higher ATP consumption rate in the kidney) and/or Explanation 2 (higher ATP production rate in the heart).

### The acidosis hypothesis for elongating hypothermic preservation time

Despite the unknown outcome of a direct kinetic comparison between hearts and kidneys, these cross-study pH trends (Figure 1B) (4, 40) strongly point to a plausible causal factor for why acceptable preservation time is shorter for the heart than for the kidney. Low pH (acidosis) would have direct negative consequences for intracellular proteins, whose structure is sensitive to [H^+^]/[OH^−^] (19, 41). Even if the heart is able to maintain intracellular pH during hypothermia, this would come at the cost of abnormally high H^+^-export activity via the Na^+^/H^+^ exchanger and other acid-sensing membrane proteins. High Na^+^/H^+^ exchanger activity causes high intracellular [Na^+^], which may trigger a cascade of events that culminate in loss of heart function (42).

Therefore, we advance the hypothesis that mitigating intracellular acidosis is key to elongating hypothermic preservation time (Figure 2). Consistent with this understanding is a study of rat heart functional recovery after 30 min of ischemia at 37°C (Figure 1C). Three pre-ischemia treatments were tested: 10 min of oxygenated perfusion, 10 min of anoxic perfusion, and 15 min of anoxic perfusion. If high ATP consumption during ischemia is desirable (in line with Explanation 1), one would predict the 10 min oxygenated group to perform best. Instead, 10 min of oxygenated perfusion prior to ischemia produced the worst recovery of heart function (in line with Explanation 2), whereas 15 min of anoxic perfusion prior to ischemia produced the best recovery. This trend aligned with post-ischemia lactate measurements, which are proportional to the degree of acidosis. For a 10 min oxygenation pre-treatment, post-ischemia lactate was highest, and therefore recovery was lowest. For a 15 min anoxia pre-treatment, post-ischemia lactate was lowest, and therefore recovery was highest (43).

The implication of this understanding is that, to improve heart 4°C preservation time, heart acidosis at 4°C should be reduced (Figure 2). A straightforward therapeutic approach, therefore, would be to inhibit heart glycolysis at 4°C. However, similar to the importance of glycolysis at 37°C (28, 44), a certain degree of glycolysis during 4°C may facilitate heart recovery.

### A balanced approach: preventing sodium overload

In line with Explanation 1, a low level of heart glycolytic ATP consumption may cause sodium overload (high intracellular Na^+^ concentration) due to low Na^+^/K^+^ pump activity (44). In line with Explanation 2, a high level of heart glycolytic ATP production may also cause sodium overload via a different mechanism, namely high Na^+^/H^+^ exchanger activity (42). In this way, both Explanation 1 and Explanation 2 point to the same preservation approach: prevent sodium overload.

A key consequence of sodium overload is increased Na^+^/Ca^2+^ exchanger activity, which in turn causes calcium overload (high intracellular Ca^2+^ concentration) (42). In a study of isolated rat hearts, high mitochondrial [Ca^2+^] was a key cause of reduced heart function, and preventing mitochondrial calcium uptake improved LVDP from 37% to 61% of initial following 45 min of ischemia at 37°C (45).

At 4°C, these membrane pumps (Na^+^/K^+^ pump, Na^+^/H^+^ exchanger, and Na^+^/Ca^2+^ exchanger) are expected to remain active, though at a much lower turnover rate than at 37°C. Therefore, the period of 37°C reperfusion is when any sodium overload (and therefore any calcium overload) is expected to become particularly pronounced.

To prevent sodium overload, one approach is to specifically inhibit Na^+^/H^+^ exchanger activity. This strategy balances two key considerations, namely (1) injury due to low ATP consumption (for which low Na^+^/K^+^ pump activity is a key mechanism) and (2) injury due to acidosis (for which Na^+^/H^+^ exchanger activity is a key mechanism). In a clinical trial (EXPEDITION) aimed at reducing cardiac injury during coronary bypass surgery, an inhibitor of the Na^+^/H^+^ exchanger, cariporide, was administered intravenously to patients before and after ischemia. This approach was efficacious in mitigating myocardial infarction (5.5% lower incidence than the placebo group), though it did not meet safety standards due to an increase in cerebrovascular events. This partial success motivates a different approach to inhibit the Na^+^/H^+^ exchanger for the application of bypass surgery (46), such as a lower dose of cariporide as has been used in more successful studies (47–49), or a different Na^+^/H^+^ exchanger inhibitor that either possesses the same monocyclic acylguanidine structure as cariporide [e.g., eniporide (50, 51)] or is more structurally distinct from cariporide [e.g., amiloride analogues (52)]. For the application of hypothermic heart storage for transplant, treatment of the donor heart with a Na^+^/H^+^ exchanger inhibitor such as cariporide followed by washout prior to transplantation would plausibly avoid the negative systemic consequence (stroke risk) observed in the EXPEDITION trial. More generally, early studies involving small mammal heart hypothermic storage are supportive of pursuing Na^+^/H^+^ exchanger inhibition for improved preservation (53, 54).

However, preventing sodium overload is downstream of acidosis, which is this article's key suggested difference between the heart and the kidney and has negative consequences beyond sodium overload (19, 41). To achieve higher heart tolerance to 4°C, it may be worthwhile to pursue parallel research to achieve a similar level of acidosis in the heart as in the kidney at 4°C.

### Learning from the kidney: preventing acidosis

Following 6 h at 4°C, ATP concentration drops to <15% of initial in the kidney yet remains at 100% of initial in the heart (Figure 1A) (10). This indicates that the kidney's ATP production rate declines more rapidly than its ATP consumption rate when transitioning from 37°C to 4°C. Therefore, to approach the kidney's level of acidosis while maintaining sufficient ATP concentration for Na^+^/K^+^ pump activity, it may be beneficial to pharmacologically inhibit some, though not all, of the heart's glycolytic ATP production at 4°C. This could be achieved by inhibiting an enzyme essential to glycolysis, such as lactate dehydrogenase (LDH), which regenerates NAD^+^—a necessary substrate for glycolysis—by converting pyruvate and NADH to lactate and NAD^+^. Interestingly, the LDH subunit that most favors the reverse reaction (lactate → pyruvate) is more abundant in the endocardium than in the epicardium (55), which correlates with our experimental results. In our study of rat hearts perfused with cryoprotective agents at 0°C, we found higher viability in the endocardium than in the epicardium (16), which may be due to less acidosis in the endocardium than in the epicardium.

Note that this strategy of reducing glycolytic ATP production is at odds with the notion that high ATP concentration (>80% of initial) at the onset of 37°C reperfusion is necessary for the recovery of heart function (10). More research would help determine whether falling below a certain ATP concentration threshold is necessarily irreversible. There is an idea that, as long as ATP precursor molecules are present––which may not be the case during acidosis, as an abnormal cytosolic [H^+^]/[OH^−^] may cause critical molecules to lose their charge and thereby diffuse out of the cell (19, 56)–the organ can reattain healthy ATP concentration if given sufficient time to recover (via machine perfusion) (19). Nonetheless, for the goal of ATP preservation—which is the ideal scenario—one good approach may be oxygenated perfusion at 4°C. By providing oxygen, ATP production via oxidative phosphorylation would increase, ideally facilitating the immediate recovery of beating upon 37°C reperfusion.

Cardiac-specific hypothermic solution design may be a beneficial alternative. UW, the hypothermic solution most commonly used in the clinic for 4°C static storage of donor hearts (57), does not contain glucose (58) and therefore does not provide a critical substrate for glycolysis. Similarly, in our study of rat hearts perfused with CPA at 0°C, we found that Celsior and UW (which do not contain glucose) substantially outperformed EC and LM5 (which do contain glucose) (16). In addition, using an effective intracellular buffer may be especially beneficial for the heart. Common buffers, ranked by their pK (capacity to accept H^+^) at 4°C, are as follows (1): HEPES (pK = 7.8), (2) histidine (pK = 7.5), (3) phosphate (pK = 6.8), and (4) bicarbonate (pK = 6.4) (19). Therefore, one strategy for improving heart recovery would be to increase the concentration of HEPES in the hypothermic perfusate and/or pre-treat the heart with high-[HEPES] perfusion at 37°C. In a study investigating the toxicity of HEPES in rats, some temporary adverse effects were observed at a dosage of 500 mg/kg (59) (∼35 mM HEPES), so safe HEPES supplementation may be below this limit. As for the cell permeability of HEPES, in a study using isolated rabbit cardiomyocytes, HEPES was shown to permeate the cell membrane and increase the cytosolic pH (60). More generally, the established 4°C storage principle of decreasing metabolism—and therefore reducing the accumulation of metabolic byproducts such as H^+^—may continue to provide a good starting point for hypothermic solution design in the heart.

### Other factors

Acidosis is likely one of multiple causal factors involved in why kidneys can recover from cold ischemia at least as well as hearts despite kidneys' substantially lower ATP concentration during cold storage. In the pathway of hypothermia-reperfusion injury, low ATP concentration and low pH can each be conceptualized as directly downstream of hypothermia whereas other events are downstream of low ATP concentration and/or low pH (e.g., low pH is a causal factor for calcium overload which is a causal factor of high levels of reactive oxygen species which is a causal factor of cell death) (19). Therefore, to provide readers with a relatively fundamental understanding, this article focused on organ-specific differences in metabolism (which cause differences in ATP concentration and/or pH). It should be noted, however, that for various downstream events in this large hypothermia-reperfusion injury pathway, there are significant organ-specific differences beyond metabolism. For example, key relevant cellular differences between the heart and kidney include a larger mitochondrial content in the heart (23) as well as the presence of Ca^2+^-containing sarcoplasmic reticula in the heart. Both of these would plausibly cause increased heart susceptibility to sodium overload damage pathways [regardless of whether this sodium overload is due to acidosis (42) and/or low ATP concentration (44)]. Nonetheless, even if heart 4°C preservation time cannot be improved to the same extent as that of the kidney, the approach of directly targeting acidosis and/or targeting the downstream effects of acidosis (e.g., sodium overload) may be a good future direction for improving 4°C heart preservation. Other good future directions may be found by consulting recent literature for other differences in heart vs. kidney that were not included in this metabolism-focused narrative review.

### Application to cryopreservation: the acidosis hypothesis of nonspecific cryoprotective agent toxicity

In a prior subsection, we advanced the acidosis hypothesis for improved heart recovery from hypothermia, which has applications including cryopreservation given that a low cryoprotective agent (CPA) perfusate temperature (e.g., 4°C) may be optimal for preventing CPA-induced injury (61)—especially at high CPA concentrations (17). In this subsection, we advance an independent acidosis hypothesis for improved recovery from CPA exposure. Future testing may reveal whether mitigating intracellular acidosis and/or its downstream effects can cause a significantly improved recovery from hypothermia alone, or a significantly improved recovery from CPA exposure (in excess of any benefit in recovery from hypothermia in the case of hypothermic CPA exposure), or neither, or both.

CPA toxicity can be subdivided into two types: specific and nonspecific (18). For specific CPA toxicity, the toxicity is due to effects specific to the CPA (e.g., dimethyl sulfoxide vs. ethylene glycol) and the intracellular molecules (e.g., aquaporin-2 vs. myosin) and can be modified by using a different CPA and/or a different cell type. For nonspecific CPA toxicity, it is not due to a specific CPA or intracellular molecule; instead, it arises from any CPA causing a general intracellular effect.

A proposed mechanism for nonspecific CPA toxicity advanced elsewhere, the qv* hypothesis, is that CPA causes nonspecific macromolecular dehydration. This hypothesis is based on data showing a negative correlation between qv* and viability. The metric qv* can be calculated for each CPA solution as the ratio of moles of water to moles of polar groups on permeating CPAs (q) at the threshold CPA concentration needed to vitrify (v) under standardized conditions (*). Accordingly, qv* can be interpreted as an individual CPA's capability for macromolecular dehydration, and the hypothesis (macromolecular dehydration) is supported by the collected data (lower viability at higher qv*) (20).

There have been concerns voiced about the dataset, including the method of calculating q (18), though nonetheless the qv* hypothesis provides data-based guidance for the design of CPA formulations for minimal nonspecific CPA toxicity. The acidosis hypothesis presented below can be conceptualized as independent of the qv* hypothesis and provides an alternate though not mutually exclusive understanding of nonspecific CPA toxicity (it is logically possible that the qv* hypothesis and the acidosis hypothesis are both true, or that one hypothesis is true and the other is false, or that both hypotheses are false).

During CPA loading, intracellular water is replaced by the permeating cryoprotective solutes, which inevitably concentrate all remaining water-soluble intracellular species, including free H^+^ and OH^−^ ions. This sudden spike in cytosolic [H^+^] skews the thermodynamic and kinetic equilibrium across the cell membrane, artificially driving high Na^+^/H^+^ exchanger activity. Similar to the mechanism of cold ischemia injury presented above, this high [H^+^] may lead to high [Na^+^], precipitating a series of events that may culminate in cell death during reperfusion (42). This may be why CPA toxicity rate increases at higher CPA concentration. In our study on CPA perfused rat hearts, for example, we were able to achieve good viability when using 41 w/v % CPA, but not 55 w/v % CPA (16). A causal factor for the proportional relation between CPA toxicity rate and CPA concentration may be that the level of dehydration and, therefore, an increase in [H^+^] is greater at higher CPA concentration. Acidosis-mediated CPA toxicity may also help explain why the CPA toxicity rate is higher at higher temperatures. In our study on CPA-perfused rat hearts, for example, we found substantially better recovery when CPA perfusion was performed at 0°C rather than 10°C (16). A causal factor for the proportional relation between CPA toxicity rate and temperature may be that the rate of glycolysis and, therefore, the degree of acidosis, is higher at higher temperatures. Finally, the acidosis hypothesis may provide insight into why certain biosystems (e.g., hearts, with higher susceptibility for acidosis) appear to be more sensitive to CPA than other biosystems (e.g., kidneys, with lower susceptibility for acidosis). A relatively controlled model system such as that of hiPSC-CMs—which undergo a metabolic shift from glycolysis to oxidative phosphorylation as they age in culture (62)—may be beneficial for testing this and other CPA toxicity hypotheses.

Altogether, the goal of this and the other analysis sections in this article is not to present a high-confidence claim of truth but to provide a hypothesis (acidosis) to guide future testing and understanding.

## Conclusion

This article explored how kidneys can recover from cold ischemia at least as well as hearts despite their substantially lower ATP concentration during cold storage. Based on our literature review of organ metabolism at 4°C and 37°C, two hypotheses were explored: (1) kidneys have a higher ATP consumption rate than hearts during hypothermia, and (2) kidneys have a lower degree and/or sensitivity to hypothermia-induced acidosis than hearts. To improve heart recovery from hypothermia, future heart preservation efforts in solution design and therapeutic interventions may benefit from targeting glycolysis and/or its downstream effects (e.g., sodium overload) at 4°C. Altogether, this article provides insight into the current state of the literature to guide future research on hypothermia preservation, with applications ranging from cold static storage to cryopreservation.
